# A severe case of hemobilia and biliary fistula following an open urgent cholecystectomy

**DOI:** 10.1186/1749-7922-4-37

**Published:** 2009-11-10

**Authors:** Vincenzo Napolitano, Roberto Cirocchi, Alessandro Spizzirri, Lorenzo Cattorini, Francesco La Mura, Eriberto Farinella, Umberto Morelli, Carla Migliaccio, Pamela Del monaco, Stefano Trastulli, Micol Sole Di Patrizi, Diego Milani, Francesco Sciannameo

**Affiliations:** 1 General Surgery and Emergency Clinic, University of Perugia S. Maria Hospital, Terni, Italy

## Abstract

**Background:**

Cholecystectomy has been the treatment of choice for symptomatic gallstones, but remains the greatest source of post-operative biliary injuries. Laparoscopic approach has been recently preferred because of short hospitalisation and low morbidity but has an higher incidence of biliary leakages and bile duct injuries than open one due to a technical error or misinterpretation of the anatomy. Even open cholecystectomy presents a small number of complications especially if it was performed in urgency. Hemobilia is one of the most common cause of upper gastrointestinal bleeding from the biliary ducts into the gastrointestinal tract due to trauma, advent of invasive procedures such as percutaneous liver biopsy, transhepatic cholangiography, and biliary drainage.

**Methods:**

We report here a case of massive hemobilia in a 60-year-old man who underwent an urgent open cholecystectomy and a subsequent placement of a transhepatic biliary drainage.

**Conclusion:**

The management of these complications enclose endoscopic, percutaneous and surgical therapies. After a diagnosis of biliary fistula, it's most important to assess the adequacy of bile drainage to determine a controlled fistula and to avoid bile collection and peritonitis. Transarterial embolization is the first line of intervention to stop hemobilia while surgical intervention should be considered if embolization fails or is contraindicated.

## Background

Percutaneous transhepatic biliary drainage (PTHBD) is one of the most therapeutic options for the menagement of biliary obstructive disorders, but the use of interventional procedures is associated with an increased incidence of arteriovenous shunting, hepatic artery pseudoaneurysm and vascular stenoses that result in hemobilia[1].

The diagnosis of hemobilia may be difficult because of a variety of clinical manifestations and sometimes can be fatal. Its management aims to stopping the bleeding and resolve obstruction. Actually the development of interventional radiology, such as transarterial embolization, has been recognized the first line of procedure to stop hemobilia with a success rate of about 80%-100%, by ensuring that the classic surgery interventions, such as ligation of bleeding vessels or excisions of aneurysms, should be considered fails and burdened by high mortality [2,3].

## Case Report

A 60-year-old man came to our observation with intermittent pain localized to upper quadrants of the abdomen, fever (39°C) preceded by thrill, vomiting and signs of peritoneal interesting. Laboratory tests revealed leucocytosis (18300 WBC), and the increment of cholestasis markers, while US scan demonstred an acute cholecystitis with lithiasis, without biliary tree dilatation, and a small liquid flap next to gallbladder.

Because of poor conditions, we decided to perform a surgical operation. So the patient underwent a laparoscopic cholecystectomy (LC) but, because of the hard consistency and the remarkable adhesions of the gallbladder to the surrounding structures (homentum, biliary tract, duodenum), we decided to stop the laparoscopic procedure and to perform an open cholecystectomy (OC). The local inflammation and gangrenous aspect of gallbladder (as the pathological report confirmed) did allow us to place a trans-cystic T-tube, to use as a biliary tutor and/or as a device, through which a cholangiography could be run, and an abdominal drainage. Post-operative clinical course progressively improved, but the T-tube flow was low (between 100-300 cc) and bilirubin level began to increase from the 5-th day after operation, while the abdominal drainage began to drain bile (500 cc).

The patient's conditions were good, without any signs of localized or generalized peritonitis or intraperitoneal bile collections: there was a controlled high flow external fistula. A conservative treatment was instituted, so the patient was nourished by parenteral way, deficits of electrolytes and vitamins (mostly vitamin K) were corrected and octreotide (somatostatin analogue) was delivered to reduce biliary secretion. Therefore we performed a trans- Kehr cholangiography to assess the origin of fistula, the anatomy of the entire biliary tree and the presence and extent of the injury to the biliary system.

**Figure 1:**
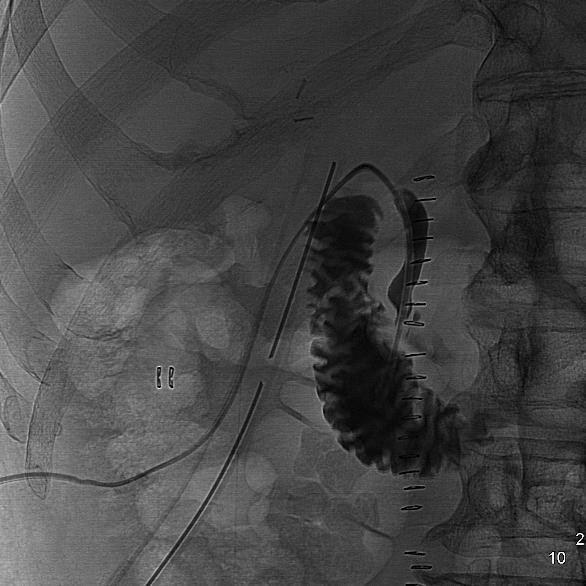
**Failure opacification of intrahepatic biliary tracts and of common biliary duct**.

**Figure 2:**
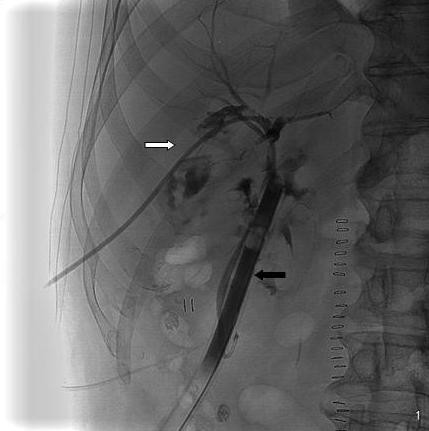
**Separation between right and left biliary ducts, abdominal drainage (black arrow), PTHBD (white arrow)**.

Cholangiography showed a separation between right and left biliary ducts, a failure opacification of intrahepatic biliary tracts and of common biliary duct because of a non complete transaction (figure 1), so we decided to position a percutaneous transhepatic biliary drainage (PTHBD) on the right biliary emisistem (figure 2) and to perform ERCP to reconstruct biliary tract.

**Figure 3:**
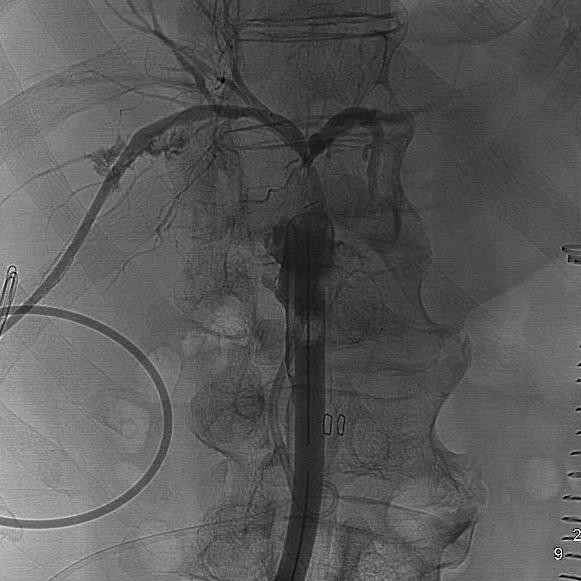
**Control: PTHBD is correctly positioned into the right biliary tract with distal tip around the surgical drainage**.

Post-operative control showed a well-positioned drainage but a biliary leakage (figure 3).

We resisted the temptation to attempt primary repair at this stage because of local inflammation. This conservative treatment was prosecuted for 3 weeks with the hope of a spontaneous closure of the fistula. But it was not so and because of the better condition of the patient, we decided to perform a new operation.

**Figure 4:**
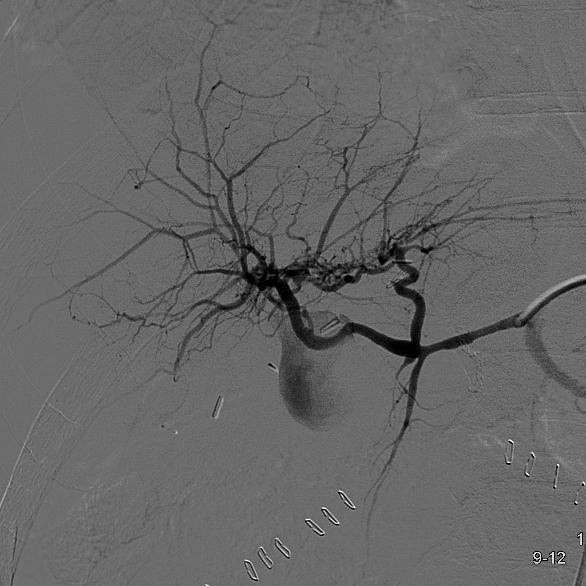
**Pseudoaneurysm on the right branch of the hepatic artery**.

**Figure 5:**
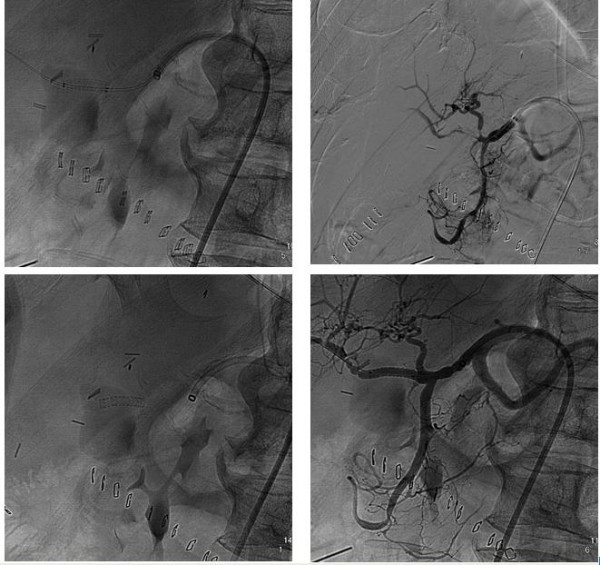
**Stenting of pseudoaneurysm; exclusion of the vascular lesion and control of the distal vascular patency. Covered stent**.

After an intra-operative cholangiography we executed an hepaticojejunostomy on left hepatic duct (the only one which was accessible) with Roux reconstruction and positioning of biliary tutor and abdominal drainage. General condition of the patient did not improve because of 3 severe episodes of cholangitis, treated with antibiotics and because a progressive anaemia. In fact on the 9-th postoperative day the patient developed a severe episode of hemobilia with abdominal type colic pain located in the epigastrium - right ipocondrium, radiating to the back and to the right shoulder, hemathemesis, jaundice and blood flow from percutaneous transhepatic biliary drainage (PTHBD), and decrement of hemoglobin levels, dropped to 7.3 g/dl. We performed an urgent volume resuscitation and contrast-enhanced CT, which showed an aspecific alteration into the V hepatic sector, so we performed a selective angiography of celiac tripode and hepatic artery that showed, on the right branch, a big pseudoaneurysm (figure 4) which was covered by stenting (figure 5).

The operative procedure was performed by right trans-femoral access and placement of a 3,5 mm × 19 mm GraftMaster Coronary covered stent (ABBOTT^®^) with total exclusion of pseudoaneurysm.

After that general conditions of the patient improved day by day and he was discharged from our unit after 45 days.

## Discussion

The management of the case reported above is very interesting because of 2 iatrogenic complications: biliary fistula and pseudoaneurysm.

Bile duct injuries and fistulas are important because they can be associated with considerable morbidity and mortality. Laparoscopic cholecystectomy is currently the standard procedure for symptomatic cholelithiasis and for all forms of cholecystitis including acute ones, even in instance of gangrenous cholecystitis. Under these difficult circumstances, the procedure is associated with an increased rate of bile duct injuries and an high conversion rate should be expected [4].

Compared with open cholecystectomy, laparoscopic cholecystectomy is associated with an increased rate of bile duct injuries ranging between 0,5-0,9% [5,6]. The mechanism of bile duct injuries are now well recognized: it's caused by misidentification of the common bile duct for the cystic duct or anomalous anatomy. After a diagnosis of biliary fistula has been made, it's most important to assess the adequacy of bile drainage to avoid bile collection and peritonitis. There are some physiopathological effects of an external biliary fistula which depend on the volume of bile drained daily with depletion of electrolytes and fluid, on the absence of bile from the gut, and on the possibility of acquired biliary infections. So a conservative treatment was made immediately: it has been known that the treatment with somatostatin can reduce bile secretion, even if its benefits in promoting closure of fistula are unproved [7].

The principles of management of postoperative biliary fistula are operative and non operative. The main goal is to drain bile collection and convert to a "controlled" fistula. When biliary-enteric continuity is present, and there is no obstruction to bile flow, a prolonged period of conservative treatment is indicated because spontaneous closure of the fistula is usual. This process can be facilitated by temporary placement of a stent across the opening in the bile duct, excluding bile flow throught the fistula as we have made in the case reported here.

Endoscopic treatment of fistula by sphincterotomy, stenting or both is indicated in most patients [8]. Often a biliary endoprothesis is used and is left in place for several weeks until fistula closure, while endoscopic sphincterotomy alone, with the intention of reducing the pressure gradient between the biliary system and duodenum, is indicated only in specific circumstances (distal biliary strictures) [9]. Operation is indicated when non operative measures are not suitable, such as in patients with diffuse bile peritonitis, in septic patients.

The increased use of interventional procedures in the management of biliary disorders is associated with an increased incidence of vascular injuries [10]. Hemobilia is an uncommon cause of gastrointestinal bleeding. Trauma has become the most common cause of hemobilia since the advent of invasive procedures such as percutaneous liver biopsy, transhepatic cholangiography, and biliary drainage; it may also be caused by infection and arteritis associated with cholecystitis or pancreatitis and shows strong associations with disease processes such as atherosclerosis, cystic medial necrosis and polyarteritis nodosa [11] but in the case reported it has been due to the presence of pseudoaneurysm of the hepatic artery.

Pseudoaneurysm accounts for nearly 10% of hemobilia cases [12], which have been associated with percutaneously placed devices [13]. Before hemobilia, we diagnosed 3 episodes of cholangitis and elevated levels of bilirubin, suggesting an increased intraductal pressure, which may have caused this vascular injury. Chronic inflammation suggests that there might be some degree of continuing low-grade damage within the liver parenchyma. As the inflammation proceeds and involves the collateral hepatic artery, a pseudoaneurysm forms and raises the risk of hemobilia. It therefore seems likely that PTHBD induced aneurismal change of the hepatic artery in combination with increased ductal pressure and cholangitis. We belive that the inflammation surrounding the bile ducts and the presence of adhesions between the PTHBD and the right branch of hepatic artery may have contributed to the formation of pseudoaneurysm because the tip of the PTHBD was at the same site of vascular injurie. Then the fistulous communication between biliary tree and vascular structures has lead hemobilia, which can be severe and life-threatening. In fact in our case reported, the patient underwent to 4 blood transfusions because of an acute anaemia and shock.

Quinkle's triad, composed by epigastric pain, hemobilia and obstructive jundice, is the classical clinical presentation of an intrahepatic artery pseudoaneurysm. These occur in 73%, 52%, and 30% of cases, respectively, although the complete triad occurred in only 22% of the them[1]. Blood may rapidly flow into the duodenum, simulating an intestinal bleeding or may lead, if the flow is slow, the formation of blood clots, obstructing the bile ducts and causing jaundice.

Our patient presented Quinkle's triad and we rapidly performed an EGDS which showed an active bleeding from ampulla of Vater, even if literature reports only a 12% of diagnostic endoscopies [14]. The choice of subsequent investigations depends on the history and the level of suspicion.

Abdominal sonography or computed tomography can detect common bile duct obstruction and identify intrahepatic lesions, such as stones or tumors.

Endoscopic retrograde cholangiopancreatography may be helpful. Angiography could detect significant hemobilia in over 90% of patients, and allow the localization of vascular lesions and therapeutic embolization.

The management of hemobilia is, in fact, aimed at stopping the bleeding and relieving biliary obstruction, especially when the condition of patient is so severe that a fast treatment is required. Transarterial embolization is now the first line of intervention to stop the bleeding of hemobilia, which returned a high success rate of around 80% to 100% [1], and lower morbidity or mortality rates than surgery. Surgical interventions, such as ligation of the bleeding vessel or excision of the aneurysm, should be considered if embolization fails or is contraindicated.

Transcatheter embolization has several advantages over surgical approaches: (a) it can be combined with angiography and also repeated, (b) it is safer because it deals directly with the arterial lesion, and (c) it is better tolerated by debilitated patients who show major surgical risks.

Treatment of these vascular lesions varies depending on the size of damaged vessels and on the characteristics of the lesions [15].

In general transcatheter embolization of distal intrahepatic vascular lesions is successfully best performed using micro-particles of a variety of materials (coil, gelatine sponge, polyvinyl alcohol, etc.) [16].

In case the pseudoaneurysm is located at the level of large hepatic vessels, the placement of a covered stent may be a valid therapeutic alternative, as we made in the case above [17-20].

On the basis of our experience, in iatrogenic hepatic bleeding, therapeutic interventional procedures represent the treatment of choice as they enable diagnosis and treatment in a single session and, especially in the case of intra-hepatic bleeding, they avoid complex surgical procedures in patients who are often haemodynamically unstable and therefore at high anaesthetic and surgical risk.

## Competing interests

The authors declare that they have no competing interests.

## Authors' contributions

VN wrote the manuscript. RC drafted the manuscript. AS revised clinical notes. LC revised clinical notes. FLM translated the manuscript into English. EF searched for the references. UM checked the patient data. CM searched for the references. PD checked the patient data. ST checked the final references list. MSDP checked the final references list. DM assessed the formatting changes. FS supervised the manuscript making. All authors have read and approved the final version of the manuscript.

## Consent Section

Written informed consent was obtained from the patient for publication of this case report and accompanying images. A copyof the written consent is available for review by the Editor-in-Chief of this journal
